# Study and QTL mapping of reproductive and morphological traits implicated in the autofertility of faba bean

**DOI:** 10.1186/s12870-022-03499-8

**Published:** 2022-04-06

**Authors:** David Aguilar-Benitez, Inés Casimiro-Soriguer, Cristina Ferrandiz, Ana M. Torres

**Affiliations:** 1 Área de Mejora y Biotecnología, IFAPA Centro “Alameda del Obispo”, Apdo. 3092 14080, Córdoba Spain; 2 Instituto de Biología Molecular y Celular de Plantas, Consejo Superior de Investigaciones Científicas – Universitat Politécnica de Valencia, 46022 Valencia, Spain

**Keywords:** Vicia faba, Autofertility, Pollen, Stigma, Flower morphology, Reproductive success

## Abstract

Autofertility describes the ability of faba bean flowers to self-fertilize thereby ensuring the productivity of this crop in the absence of pollinators or mechanical disturbance. In the legume crop faba bean (*Vicia faba* L.), lack of autofertility in a context of insufficient pollination can lead to a severe decrease in grain yield. Here we performed the first QTL analysis aimed at identifying the genomic regions controlling autofertility in this crop. We combined pod and seed setting scores from a recombinant inbred population (RIL) segregating for autofertility in different environments and years with measurements of morphological floral traits and pollen production and viability. This approach revealed 19 QTLs co-localizing in six genomic regions. Extensive co-localization was evident for various floral features whose QTLs clustered in chrs. I, II and V, while other QTLs in chrs. III, IV and VI revealed co-localization of flower characteristics and pod and seed set data. The percentage of phenotypic variation explained by the QTLs ranged from 8.9 for style length to 25.7 for stigma angle. In the three QTLs explaining the highest phenotypic variation (*R* ^2^ > 20), the marker alleles derived from the autofertile line Vf27. We further inspected positional candidates identified by these QTLs which represent a valuable resource for further validation. Our results advance the understanding of autofertility in faba bean and will aid the identification of responsible genes for genomic-assisted breeding in this crop.

**Supplementary Information:**

The online version contains supplementary material available at 10.1186/s12870-022-03499-8.

## Background

With a global production of 5.4 million metric tons, faba bean (*Vicia faba* L.) is the fourth most widely grown cool season legume after chickpea (*Cicer arietinum*), pea (*Pisum sativum*), and lentil (*Lens culinaris*) [1]. The crop is grown across a wide agro-geographical area in Asia, Africa, Middle East and Australia and used for both animal and human consumption. This legume is characterized by a high protein content and high-yield potential and thus plays an important role as a food and feed legume crop. In addition, faba bean contributes to soil fertility through nitrogen fixation when used as a diversification crop in rotations. However, although faba bean represents an important legume crop, the large size of its genome (13Gbp) and the lack of a complete reference genome sequence limit the use of molecular tools and the identification of candidate genes in genomics-assisted breeding for agronomic traits of interest.

Potential yield in faba bean is highly variable. Apart from biotic and abiotic stresses, productivity and yield stability across generations are impacted by the partial allogamy of the species which varies between cross- and self-pollination [2]. Cross-pollination depends on pollinators such as honey bees, solitary bees or bumblebees and low visitation rates have been related to unstable yields as well as to lower pod and seed set [2–5]. Self-pollination may also require a pollinator visit to manipulate the flower (tripping). The rate of cross-fertilization ranges between 4 and 84% (mean 30–60%) and mainly depends on genetic and environmental factors [6–8]. Insufficiency of pollination can act as a major constraint to potential yield [2, 9].

Autofertility is defined as the ability of a flower to self-fertilize in the absence of insect pollinators or mechanical disturbance [10]. In faba bean two types of autofertility have been described: ‘heterotic autofertility’ in F1 hybrids which results from outcrossed plants and ‘fixed (or additive) autofertility’ in inbreds [2]. The degree of autofertility differs among faba bean genotypes [7, 11, 12]. F1 hybrids are usually more autofertile than inbred genotypes, doubling autofertility over the parents [7].

Although cross-pollination improves yield stability and resistance to biotic and abiotic stresses [13–18], selection for autofertility could ensure pod and seed set in the absence of pollination services [11, 19, 20]. Previous studies used different parameters to study the autofertility, such as number of seeds per flower, pods per plant and seeds per plant [10, 21]. Stoddard [22] applied complex parameters to describe autofertility, with incidence and effectiveness of pollination, incidence of fertilization in flowers and ovules, and index of fertilization. Link [7] simplified the autofertility analysis as seed containing pods per standardized number of untripped flowers. More recently, Puspitasari [23] quantified the level and variation of autofertility in winter faba bean breeding germplasm and performed association analysis for different traits (e.g. first flower position, flowering time, plant height, seed yield and thousand kernel weight). Although significant correlations between some agronomic and autofertility-related traits were identified, no significant QTLs could be found.

Selection for high or low levels of autofertility are accompanied by changes in floral traits functionally related to pollinators, and these changes are generally in opposite directions [24, 25]. Different flower features have been found to be related with autofertility in faba bean including the quality and quantity of pollen, the style-ovary angle, the length of papillae on the stigma, the thickness or hardness of the cuticle that retains the stigmatic exudate, and the amount of that exudate [19, 26, 27].

While the need of the exudate release for pollen germination and further steps towards fertilization is self-evident, the importance of some of these features are still unclear. Kambal et al. [19] reported that inbred lines with the ability to spontaneously self-pollinate showed a wider style-ovary angle, shorter style, few and short stylar hairs and stigmatic papillae and smoother ridges on the inside of the keel petals. Regarding the amount of pollen, F1 hybrids produced more pollen grains than the parental lines, however they found an association between autofertility and lower quantities of pollen in inbred lines. Lord and Heslop [28] also found that the autofertile line used in their work had low stigma papillae at the receptive tip, with a relatively thin intervening cuticle, while the autosterile line had longer papillae and a thicker cuticle. A line with partial autofertility was intermediate in these characteristics. A more recent work by Chen [27], reported as well a wider style-ovary angle, few and shorter stigmatic papillae and more pollen production in the autofertile groups, but longer styles in the autofertile lines. She also detailed that the release of exudate due to the rupture of the stigmatic cuticle was previous to anthesis in autofertile lines. Apart from the floral design traits, other studies have associated water limitation or heat stress with a reduction of the autofertility in faba bean [22, 28–30].

Despite the number of studies on autofertility and co-segregating floral traits carried out previously, there is still limited knowledge on the molecular basis underpinning these features, on which floral traits or reproductive parameters are involved in the response to selection for autofertility and what is their location in the faba bean genetic map. Here we performed a comprehensive analysis of autofertility in faba bean, by (i) characterizing different traits associated with faba bean pod and seed setting in a recombinant inbred population (RIL) segregating for autofertility in different environments (under insect-proof cages and open field) and years, (ii) evaluating components of flower structure that are thought to affect autofertility such as quality and quantity of pollen, style-ovary angle, length of stigmatic papillae etc., and (iii) performing for the first time a QTL analysis to localize chromosomal regions that significantly affect the variation of these traits in the RIL population. These results add to the understanding of autofertility in faba bean and will help in the identification of the responsible genes for genomic-assisted breeding.

## Results

### Field phenotypic evaluations

The number of flowers per node (FN), number of pods per node (PN), pod set (PS), ovules per ovary (OV), seeds per pod (SP) and seed set (SS) recorded in parental lines and RILs for each agronomic season are shown in Table 1. Considering all the agronomic seasons, the mean number of flowers per node was much higher for Vf6 (7.65 flowers ± 0.46; mean ± 1SE) than for Vf27 (1.29 flowers ± 0.07), whereas the mean number of pods per node was similar (0.65 pods ± 0.10 for Vf6 and 0.61 pods ± 0.09 for Vf27). Consequently, the pod set was much higher in line Vf27 (49.75% ± 8.66) than in Vf6 (8.28% ± 0.92). Regarding ovules and seeds, Vf6 showed a lower number (2.41 ovules ± 0.38; 1.88 seeds ± 0.40) than Vf27 (3.02 ovules ± 0.12; 2.93 seeds ± 0.11) while the seed set was higher in Vf27 (97.22 ± 0.81) than in Vf6 (77.74 ± 7.99). Differences in pod set were significant between parental lines (*p* < 0.001) and environments (*p* < 0.05), however seed set was only significant between environments (*p* < 0.05), with higher values for pod and seed set in open field conditions (Additional file 1).

**Table 1:** Mean ± 1SE values for flowers per node (FN), pods per node (PN) and pod set (PS), ovules per ovary (OV), seeds per pod (SP) and seed set (SS), for each agronomic season and environment. The environment is indicated by C (insect-proof cages) or F (open field)

	**FLOWERS PER NODE (FN)**					**PODS PER NODE (PN)**					**POD SET (%) (PS)**				
	**Vf6**		**Vf27**		**RIL population**	**Vf6**		**Vf27**		**RIL population**	**Vf6**		**Vf27**		**RIL population**
	**Mean**	**1SE**	**Mean**	**1SE**	**Range** **(min–max)**	**Mean**	**1SE**	**Mean**	**1SE**	**Range** **(min–max)**	**Mean**	**1SE**	**Mean**	**1SE**	**Range** **(min–max)**
2008_2009C	6.67	0.50	1.38	0.14	1.70 – 6.74	0.53	0.11	0.83	0.05	0.00 – 2.00	7.78	1.70	64.25	6.41	0.00 – 75.00
2009_2010F	-	-	-	-	2.22 – 8.40	-	-	-	-	0.00 – 2.40	-	-	-	-	0.00 – 63.80
2009_2010C	8.76	0.20	1.19	0.08	2.36 – 9.52	0.63	0.08	0.66	0.15	0.18 – 2.94	7.10	0.85	59.63	14.28	3.10 – 63.50
2010_2011F	6.93	0.84	1.21	0.09	1.75 – 8.02	0.73	0.28	0.37	0.18	0.00 – 2.31	9.25	3.29	27.33	12.78	0.00 – 56.50
2012-2013F	8.81	0.21	1.17	0.04	1.89 – 6.95	0.99	0.09	0.78	0.04	0.64 – 2.33	11.29	0.94	67.31	3.43	10.29 – 77.14
2014_2015C	7.10	0.23	1.54	0.08	1.92 – 7.88	0.42	0.10	0.45	0.09	0.06 – 1.82	6.00	1.48	30.21	5.87	1.39 – 57.60
	**OVULES PER OVARY (OV)**					**SEEDS PER POD (SP)**					**SEED SET (%) (SS)**				
	**Vf6**		**Vf27**		**RIL population**	**Vf6**		**Vf27**		**RIL population**	**Vf6**		**Vf27**		**RIL population**
	**Mean**	**1SE**	**Mean**	**1SE**	**Range** **(min–max)**	**Mean**	**1SE**	**Mean**	**1SE**	**Range** **(min–max)**	**Mean**	**1SE**	**Mean**	**1SE**	**Range** **(min–max)**
2008-2009C	2.66	0.23	3.24	0.09	1.99 – 3.77	1.46	0.1	3.08	0.1	1.00 – 3.32	56.22	3.37	95.22	1.66	31.25 – 98.39
2009-2010C	2.48	0.12	2.68	0.14	1.70 – 3.75	2.24	0.1	2.62	0.14	1.70 – 3.63	90.64	2.12	97.72	0.97	74.92 – 100.0
2009-2010F	1.33	-	3.11	0.23	1.78 – 3.51	1.00	-	3.00	0.19	1.70 – 3.29	75.00	-	92.11	1.04	77.28 – 100.0
2012-2013F	3.15	0.08	3.06	0.13	1.99 – 4.14	2.80	0.09	3.03	0.12	1.72 – 4.06	89.11	1.72	99.10	0.65	75.53 – 100.0

**Fig. 1:**
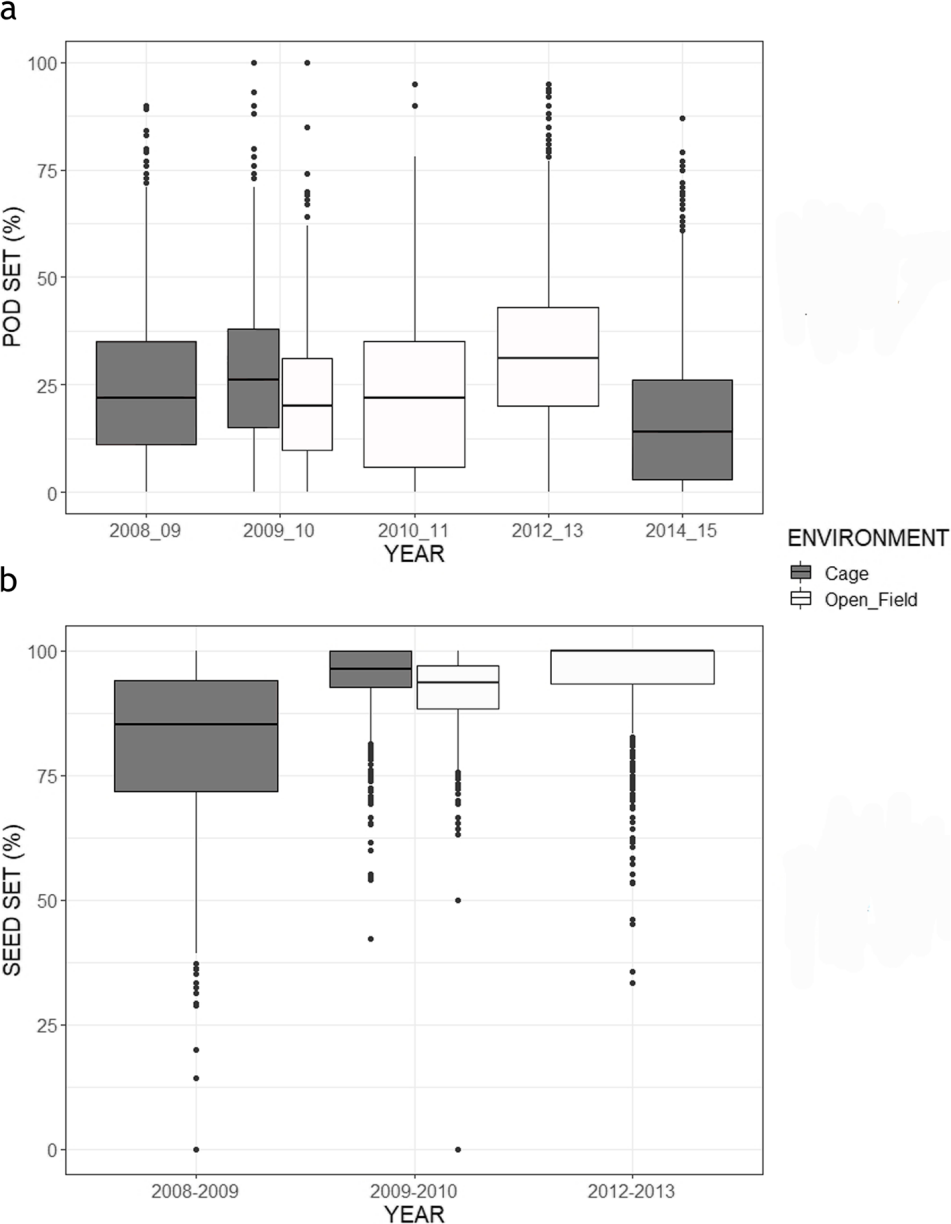
**a** Pod set and (**b**) seed set values for the whole population in different years. Assays under insect proof cages are shown in grey while assays in open field condition are shown in white. Boxes represent 25 and 75 percentiles, central solid lines are median values

Taking into account all the data and years evaluated in the whole RIL population, the statistical analysis showed no significant differences in pod set or seed set between environments (open field vs. insect-proof cages), when only environment was included as a fixed factor (results not shown). However, when the year was also considered, the model showed significant differences between the two environments and years (Table 2; Fig. 1). Specifically, pod set in seasons 2012–2013 and 2014–2015 was significantly different from 2008 to 2009, whereas 2009–2010 and 2010–2011 were similar to 2008–2009 (Table 2; Fig. 1). Seed set in seasons 2009–2010 and 2012–2013 significantly differed from season 2008–2009.

**Table 2:** Result from the GLMMs testing the effect of environment and year on pod and seed set. The model was fitted using repeats nested within the environment and year as a random factor. Parameter estimates for the level of fixed factors were calculated using “cage” as reference for the variable environment and “2008_09” for the variable year

	**POD SET**			**SEED SET**		
	**Estimate ± SE**	**z**	**P**	**Estimate ± SE**	**z**	**P**
**Environment**						
Open field	-0.31 ± 0.13	-2.37	0.018 *	-0.8 ± 0.29	-2.75	0.006 **
**Year**						
2009_10	0.17 ± 0.11	1.52	0.128	2.25 ± 0.27	8.27	< 0.001 ***
2010_11	0.27 ± 0.17	1.58	0.114	-		-
2012_13	0.71 ± 0.17	4.22	< 0.001 ***	2.68 ± 0.38	6.97	< 0.001 ***
2014_15	-0.50 ± 0.11	-4.51	< 0.001 ***	-	-	-

In 2009–2010 both conditions were assayed (open field vs. insect-proof cages). The statistical analysis revealed significant differences between environments for pod (*P* < 0.001) and seed set (*P* < 0.05), with higher values for both traits in plants grown under the insect-proof cages as compared to those grown in the open field (Fig. 1).

### Pollen size and production

Total pollen production (TOTALQ, mean ± 1SE) showed significant differences between the parental lines (*P* < 0.01) with more than double grains per flower in Vf6 compared to Vf27 (22,837 ± 2,011 vs. 11,184 ± 1,859). Mean pollen production of RILs was 19,102 ± 491 with a minimum of 8,432 pollen grains (RIL75) and a maximum of 32,744 pollen grains (RIL03).

The mean size of the pollen grains in the whole range (TOTALS) differed significantly between parental genotypes (*P* < 0.01; 22.4 ± 0.5 μm for Vf6 vs. 30.1 ± 0.4 μm for Vf27). The mean size of the pollen grains for the RILs was 26.9 ± 0.3 μm, with a minimum of 20.0 μm (RIL16) and a maximum of 32.6 μm (RIL77).

**Fig. 2:**
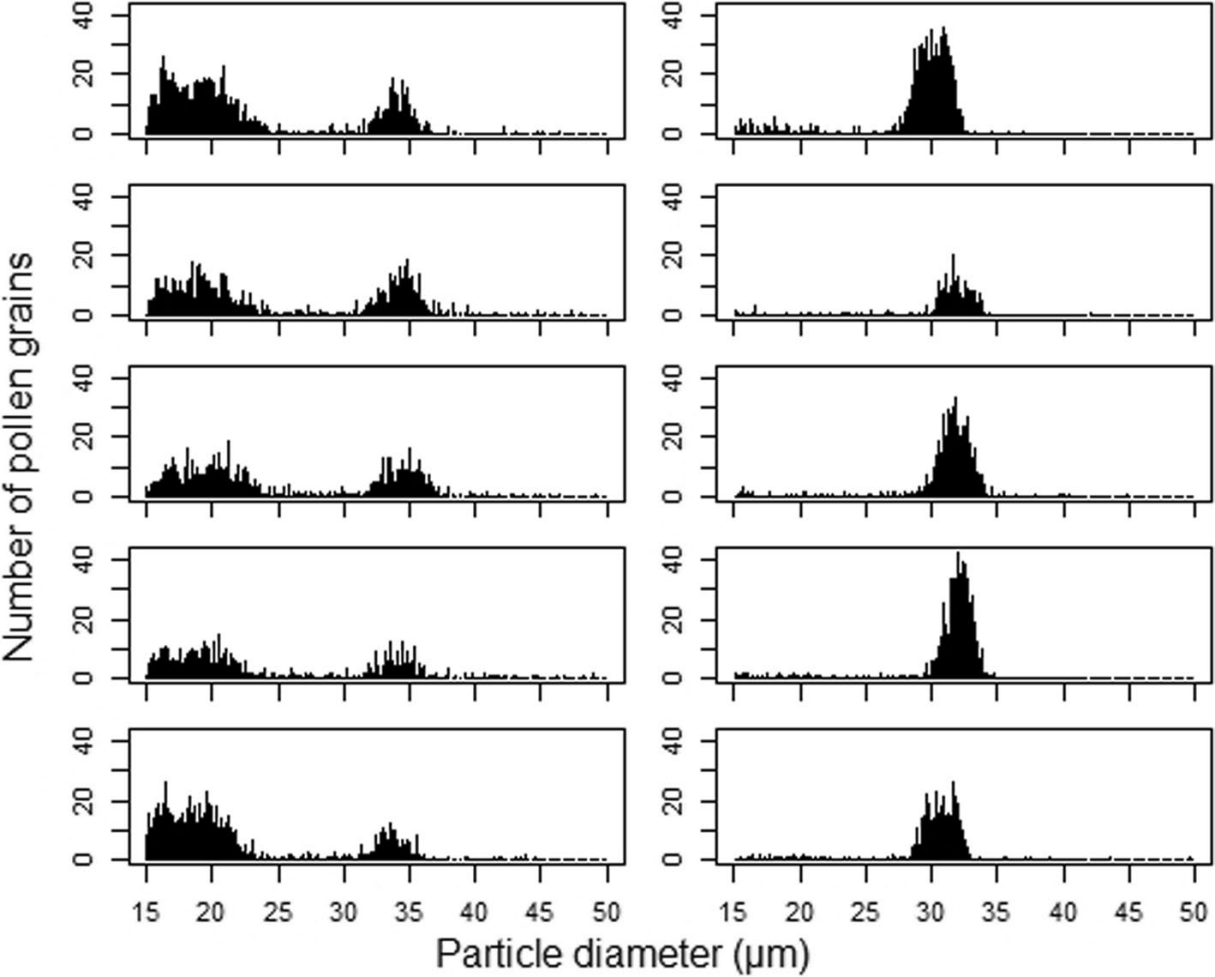
Number of pollen grains by particle size in Vf6 (left) and Vf27 (right)

Since the equatorial pollen size in faba bean has been reported as ~ 30 μm [31], we asked why some lines showed lower mean values. Figure 2 shows the number of pollen grains found for each particle size in five samples from parental lines Vf6 and Vf27. Both lines showed a peak in the number of pollen grains with the expected size (27 ~ 38 μm, hereafter designated normal pollen), but Vf6 also showed a large number of pollen grains with smaller size (hereafter named abnormal pollen).

To estimate the production and size of normal pollen in all samples, we restricted the size to 27–38 μm. In this range, pollen quantity (NORMALQ) was also different between parental lines (*P* < 0.01). Vf27 had more than twice normal pollen (10,100 ± 1,644 pollen grains) than Vf6 (4,934 ± 308 pollen grains). Mean normal pollen production of the RILs was 11,001 ± 527 with a minimum of 1,676 (RIL117) and a maximum of 24,656 (RIL02) pollen grains. Normal pollen size (NORMALS) also showed significant differences between the parental lines (*P* < 0.001) with larger sizes in Vf6 (33.5 ± 0.2 μm) than in Vf27 (31.3 ± 0.4 μm). Mean pollen size of the RILs was 32.6 ± 0.05 μm.

### Pollen viability and stigma receptivity

The GLMM confirmed the results found in Fig. 2 showing significant differences in the proportion of viable pollen between the parental lines (*P* < 0.001), with a higher proportion of non-viable pollen in Vf6 compared to Vf27. No differences were observed in pollen viability between the two flower sizes analysed. However, the interaction between line and flower size (type) was significant (*P* < 0.001) indicating that pollen viability in Vf6 is lower in larger flowers (Additional file 2). On the other hand, the two-way ANOVA analysis showed differences in pollen size between parental lines and pollen types (*P* < 0.001). This means that Vf6 has a larger pollen size than Vf27, and that in both parental lines viable pollen grains were significantly larger than non-viable ones (Additional file 3**, **Additional file 4).

Stigma receptivity staining in the four developmental stages flower bud, pre-anthesis, complete anthesis and complete anthesis after a tripping treatment revealed that the stigma in the parental line Vf27 was receptive (stained) in almost all samples from the four developmental stages whereas in Vf6 the stigma remained largely unstained (Additional file 5). Only three out of ten stigmas of flowers in complete anthesis were stained after the tripping treatment (data not shown).

### Morphological measures of the flower, ovary and style

Flower, ovary and style lengths were significantly higher in Vf6 than in Vf27 (Table 3), although apex length was similar in both lines. Since flower length differed significantly, we decided to standardize all morphological traits to this trait in order to eliminate differences caused by different flower size. Statistical analysis detected no variation between the standardized measures, indicating that the flower morphology is proportionally similar between the parental lines.

The most interesting trait was the style-ovary angle (SOA), which displayed significant differences between genotypes, with Vf6 showing sharper angles (~ 79°) than Vf27 (~ 96°). In the RIL population, the most acute angle was in RIL119 (75.65°) while the most obtuse angle was in RIL14 (100.77°).

**Table 3:** Mean ± 1SE values for flower length (FL), ovary length (OL), style length (SL), apex length (AL), style-ovary angle (SOA) and their normalization to flower length for Vf6, Vf27 and the RIL population. Asterisks indicate significant differences between parental lines (** *P* < 0.01, *** *P* < 0.001)

	**VF6**			**VF27**			**RIL population**	
	**N**	**Mean**	**1SE**	**N**	**Mean**	**1SE**	**Range (Min–Max)**	**Mean ± 1SE**
FL**	22	24.02	0.32	11	22.27	0.36	22.19 – 25.65	23.80 ± 0.05
OL***	22	14.67	0.14	11	13.41	0.18	12.65–15.33	14.24 ± 0.05
SL***	22	3.93	0.03	11	3.63	0.03	3.28–4.40	3.84 ± 0.02
AL	22	1.42	0.03	11	1.37	0.03	1.09–1.72	1.35 ± 0.01
SOA***	22	79.24	0.54	11	95.63	1.13	75.65–100.77	85.69 ± 0.45
OL/FL	22	0.61	0.00	11	0.60	0.00	0.55–0.66	0.60 ± 0.002
SL/FL	22	0.16	0.00	11	0.16	0.00	0.15–0.18	0.16 ± 0.001
AL/FL	22	0.06	0.00	11	0.06	0.00	0.04–0.07	0.06 ± 0.00

### Stigma measures using scanning electron microscopy

The stigma length and stigma area were significantly larger for Vf6 than for Vf27 (*P* < 0.01 and *P* < 0.001, respectively). However, no differences were found for the parameters Rupture length (RUPTL) and Ruptured area (RUPTAREA) or the percentages thereof (Table 4). The number, length and width of papillae in Vf6 was significantly higher than in Vf27, although PAPLW_RATIO and the number of papillae divided by stigma length (NPAP/STIGL) was similar. Density of papillae (PAPD) was significantly higher for Vf6 than for Vf27 (*P* < 0.001). Moreover, stigma angle also differed significantly between the parental lines, with wider angles in line Vf6 than in Vf27 (*P* < 0.001, Table 4).

**Table 4:** Mean ± 1SE values for the stigma length (STIGL), rupture length (RUPTL), percentage of rupture (%RUPT), stigma area (STIGAREA), ruptured area (RUPTAREA), percentage of ruptured area (%RUPTAREA), number of papillas (NPAP), papilla length (PAPL), papilla width (PAPW), ratio papilla length/width (PAPLW_RATIO), number of papilla by stigma length (NPAP_STIGL), papilla density (PAPD) and stigma angle (STIGA) for Vf6, Vf27 and the RIL population. Asterisks indicate significant differences between parental lines (* *P* < 0.05, ** *P* < 0.01, *** *P* < 0.001)

	**VF6**		**VF27**		**RIL population**	
	**Mean**	**SE**	**Mean**	**SE**	**Range**	**Mean ± 1SE**
Stigma length (STIGL)**	226.14	4.59	200.26	5.71	144.36 – 248.94	195.74 ± 1.94
Rupture length (RUPTL)	22.49	6.99	33.04	5.91	0 – 89.42	18.97 ± 1.86
Percentage of rupture (%RUPT)	10.09	3.18	16.76	2.90	0 – 38.07	9.35 ± 0.86
Area stigma (STIGAREA)***	56,350.69	1755.11	40,248.23	1110.68	21,127.9 – 69,665.6	40,166.6 ± 769.7
Ruptured area (RUPTAREA)	3158.60	1167.04	2540.60	987.49	0 – 9648.4	1793.0 ± 189.8
Percentage of ruptured area (%RUPTAREA)	5.92	2.26	6.53	2.58	0 – 25.5	4.5 ± 0.46
Number of papilla (NPAP) ***	13.29	0.36	10.17	0.31	7 – 14.75	11.3 ± 0.11
Papilla length (PAPL)*	12.94	0.50	11.58	0.36	7.60 – 14.50	10.81 ± 0.12
Papilla width (PAPW)*	8.08	0.17	7.40	0.23	4.85 – 8.98	7.03 ± 0.07
Papilla length/width ratio (PAPLW_RATIO)	1.60	0.05	1.57	0.03	1.24 – 2.16	1.52 ± 0.01
Number papilla/STIGL (NPAP_STIGL)	0.06	0.00	0.05	0.00	0.04 – 0.07	0.0590 ± 0.00
Papilla density (PAPD)***	24.44	0.67	20.11	0.79	19.6 – 35.0	26.9 ± 0.28
Stigma angle (STIGA)***	64.79	2.05	48.49	0.67	33.75 – 73.40	54.1 ± 0.71

### QTL analysis

**Fig. 3:**
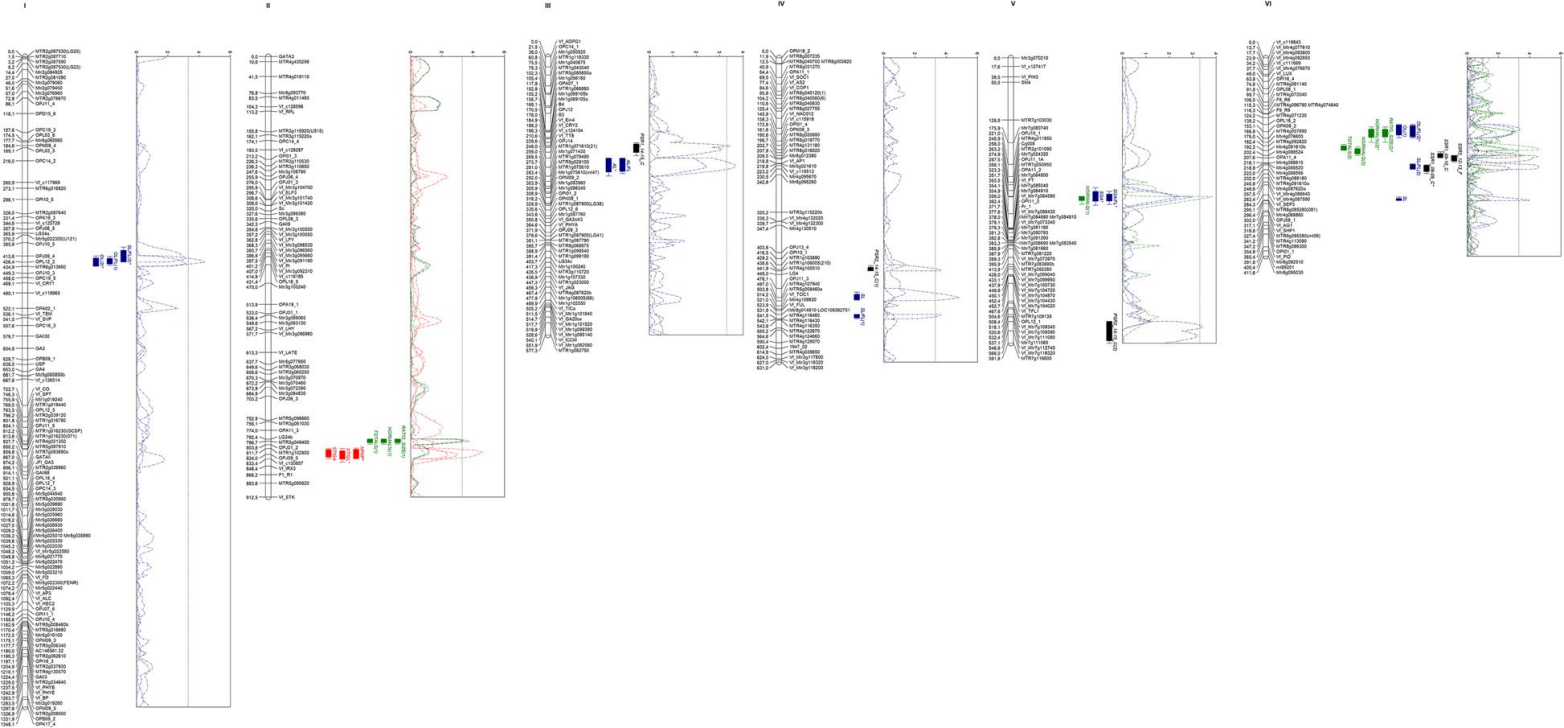
Linkage map and QTLs for autofertility traits detected in the Vf6 x Vf27 RIL population. QTL locations are represented by bars (2-LOD interval) and boxes (1-LOD interval). Black traits: field measures; green traits: pollen measures; blue traits: morphological measures and red traits: stigma measures. OL/FL: ovary length/flower length; SL/FL: style length/flower length; OL: ovary length; RATIO_SIZE: ratio normal size/total size; NORMAL%: percentage of normal pollen; TOTALS: mean size of the pollen grains in the whole range; NPAP: number of papilla; STIGL: stigma length; STIGA: stigma area; PSR1_14–15_C: pod set repeat 1 in cage; AL/FL: apex length/flower length; AL: apex length; PSR2_14–15_C: pod set repeat 2 in cage; SL: style length; SOA: stigma-ovary angle; SOA/FL: stigma-ovary angle/flower length; NORMALQ: pollen quantity of normal size; SSR1_08–09_C and SSR1_09–10_C: seed set repeat 1 in cages; SSR1_12–13_F: seed set repeat 1 in field

Our analysis detected 19 significant QTLs (LOD threshold > 3.3), five of which are located in chrs. III, IV, V and VI and correspond to pod or seed set in cages or open field assays (Fig. 3; Table 5). In addition, six QTLs for pollen size and production are located in chrs. II, V and VI; seven for morphological flower measures are in chrs. I, III, IV and VI; and one for SEM measures is in chr. II. Several putative QTLs did not reach the level of significance and co-localized with significant QTLs for the same trait (Fig. 3, Additional file 6). Twelve of the QTLs showed positive additive effects, indicating that the alleles originate from the female parent Vf6. In the remaining seven QTLs, the alleles derive from the autofertile line Vf27 and some of them explained the highest phenotypic variation (e.g. STIGA, AL/FL, SSR2_12–13_F and TOTALS).

**Table 5:** Significant QTLs detected in the Vf6 x Vf27 RIL population. No. 1–5: Pod set (PS) and seed set (SS) QTLs from different field phenotypic evaluations. No. 6–11: QTLs for pollen measures; No. 12–18: QTLs for flower structures and No. 19: QTL for stigma characteristics. R1: repeat 1; R2 Repeat 2; C: insect-proof cages, F: open field evaluations. Number between brackets indicates the different QTLs detected for the same trait. ^a^Chromosome position of the QTL marker; ^b^Chromosome. In bold, significant markers closest to the LOD peak and falling within the 2-LOD QTL interval

	**No**	**QTLs**	**Position**^**a**^	**Chr**^**b**^	**Flanking Markers**	**LOD**	**Additive effects**	***R***^***2***^
**Field evaluations**	1	PSR1_14-15_C	184.859	III	**Vf_Ein4**/Vf_TT8	3.44	0.052	14.1
	2	PSR2_14-15_C(1)	439.630	IV	**MTR1g106005(210)**/MTR4g100510	3.64	-0.048	13.2
	3	PSR2_14-15_C(2)	557.567	V	**Vf_MTR7g112740/Vf_MTR7g118320**	3.41	0.056	18.2
	4	SSR1_09-10_C	202.410	VI	**Mtr4g088524**/OPA11_4	3.69	-0.028	15.8
	5	SSR2_12-13_F	204.410	VI	**Mtr4g088524/OPA11_4**	4.52	-0.025	22.4
**Pollen measures**	6	RATIO_SIZE(1)	795.357	II	**LG34b/MTR3g049400**	3.34	0.035	11.5
	7	NORMAL%(1)	795.357	II	**LG34b/MTR3g049400**	3.36	8.172	11.3
	8	TOTALS(1)	796.735	II	**MTR3g049400**/OPJ01_2	3.75	1.233	13.3
	9	NORMALQ(1)	289.096	V	**MTR7g050950**/OPJ11_1A	3.83	24.840	9.4
	10	TOTALS(2)	189.393	VI	**Mtr4g092820/Mtr4g091610b**	4.60	-1.433	17.6
	11	NORMALQ(2)	192.178	VI	**Mtr4g091610b**/Mtr4g88524	3.32	-18.515	10.0
**Morphological measures**	12	OL/FL(1)	426.395	I	OPJ09_4/**OPL12_2**	4.36	0.010	14.8
	13	AL/FL	226.615	III	**Vf_TT8/OPJ14**	3.68	-0.002	20.1
	14	SL(1)	496.976	IV	**MTR4g107940**/MTR5g008460a	4.82	0.062	13.2
	15	SL/FL(1)	532.928	IV	**LOC109362751**/MTR4g116460	3.64	0.003	12.5
	16	OL(1)	147.245	VI	**OPL18_2/OPK09_2**	3.39	0.226	14.5
	17	SL/FL(2)	226.008	VI	Mtr4g088615/**Mtr4g088595**	4.58	0.003	13.2
	18	SL(2)	295.070	VI	Vf_SEP3/**Mtr8g085280(81)**	4.00	0.056	8.9
**Stigma measures**	19	STIGA	819.677	II	**MTR1g102900/OPJ09_5**	4.59	-0.027	25.7

Four out of five QTLs for yield related traits were evaluated in insect-proof cages including pod set (PS) 2014–2015 and one of the seed set QTLs (SS) 2009-10. The QTLs for PS are located in chrs. III, IV and V, explaining between 13.2 and 18.2% of the phenotypic variation, while the SS QTL in chr. VI (SSR1_09–10_C) accounts for 15,8% of the variation. Interestingly, a second QTL for SS evaluated in the open field in 2012-13 co-localized with SSR1_09–10_C and with most relevant QTLs for flower and pollen traits (see below).

QTLs for pollen measures revealed two important genomic regions in chrs. II and VI. Three QTLs for the pollen traits RATIO_SIZE(1), NORMAL%(1) and TOTALS(1), co-localized at the same position in chr. II around markers LG34b and Mtr3g049400, explaining between 11.3 and 13.3% of the phenotypic variation. In chr. VI, QTLs for NORMALQ(2) and TOTALS(2) were closely linked and explained 10 and 17.6% of the variation, respectively, while in chr. V, a QTL for NORMALQ(1) explained 9.4% of the variation. Regarding the morphological measures of the flower, ovary length (OL), apex length (AL), style length (SL) and their normalization revealed significant QTLs spread over four chrs., explaining between 8.9 and 20.1% of the phenotypic variation. The single QTL for SEM measures (STIGA, angle of stigma) detected in chr. II explained the highest percentage of variation (25.7%) (Fig. 3; Table 5).

In addition, we found 11 non-significant QTLs that co-localized with one or several significant QTLs, including one QTL for field evaluation, two for pollen traits, six for morphological measures and two for stigma traits measured under the SEM (Additional file 6). QTLs for morphological traits (SL/FL, OL, AL and OL/FL) co-localized with other morphological measurements in chrs. I, III and VI, whereas the remaining two QTLs, SOA/FL and SOA, co-localized with the pollen trait NORMALQ in chr. V. The non-significant QTLs for pollen traits co-localized with the morphological trait OL in chr. VI, while the QTLs for stigma traits NPAP and STIGL co-localized with the significant QTL STIGA in chr. II. Finally, SSR1_08–09_C co-localized in chr. VI with the QTL for morphological measure SL/FL(2).

A BLASTp search was performed using the sequence database of the model plant *Arabidopsis thaliana*, to identify the orthologs of the genes flanking each QTL (Additional file 7). Interestingly, some of these genes (Vf_Ein4, MTR1g106005(210), Mtr4g088595, MTR3g049400, MTR7g050950 and MTR1g102900) have been related to pollen and reproductive organs development in different species over the last years (see details in Discussion) thus, suggesting that candidate genes identified in the current work have great potential to modulate directly or indirectly the autofertility in this crop.

## Discussion

Global climate change is a major challenge of this century, with higher temperatures and CO_2_ concentrations and alteration of rainfall regimes among the most important parameters [32]. One of the key agricultural processes affected by high temperature is plant reproductive success [30, 33], either directly through physiological alterations of crucial development stages [34], or for entomophilous plants, indirectly through disruption of plant–pollinator interactions [35]. For example, climate change can cause the uncoupling of insect cycles and flowering phenology [36] or produce changes in the population distribution [37]. Faba bean breeding is slow and costly due to its common mixed self- and outcrossing habit and lack of autofertility. Therefore, understanding the traits that promote autofertility and identifying genomic locations controlling this trait is of great importance in breeding programs.

Although the parental line Vf6 produces a higher number of flowers and a similar number of pods per node than Vf27, the final pod set is higher in Vf27. This finding, together with the higher values of seed set implies that Vf27 has a higher reproductive success than Vf6. In this study, the analysis of the pod and seed sets in the whole RIL population in different years and conditions revealed important fluctuations that further confirmed the significant effect of the environment on yield components. Interestingly, the single year analyzed in both conditions revealed higher pod and seed set values in the absence (cages) compared to the presence of pollinators (open field). Recently, Bishop et al. [38] reported that pollination treatments generally improved yield, although in certain cultivars yield was lower with additional pollination. Pollination dependence also varied between growth conditions (insect-proof cages or open field assays), years as well as other parameter used for yield evaluation.

Regarding pollen production, the amount of pollen detected in the parental lines and RILs (Vf6: 22,837; Vf27: 11,184; RILs: 19,102 pollen grains) is similar to that found in other studies [27, 39], although it differs from that reported by Kambal et al. [19]. Regarding pollen size, the equatorial width of normal pollen in this study (~ 33 μm) is similar to that reported (~ 30 μm) by Poulsen and Martin [31], but larger than that described by Kurkina et al. [40] (24.5 μm). This indicates a certain variability in the pollen size depending on the crop variety. Another interesting result emerging from our analysis is that line Vf6 produced a high number of pollen grains, but a large proportion thereof (> 70%) showed a smaller size than that expected in regular pollen. In addition, viability tests showed that the smaller pollen grains were non-viable. Therefore, in spite of a lower total pollen production, the quantity and viability of the normal pollen in line Vf27 was higher than in Vf6. Similarly, Chen [27] found that the F1 hybrids and autofertile segregants of the cross between K25 (autofertile) and D07 (autosterile) showed higher pollen production than the autosterile lines. The asynaptic nature of line Vf6 could be the cause for the large fraction of non-viable pollen. Several studies with asynaptics lines of *V. faba* [41] and *Solanum nigrum* [42] revealed a higher amount of non-viable pollen.

Our stigma receptivity tests showed that the stigma tip of the autosterile line Vf6 remained unstained even in anthesis and only some flowers were stained after tripping. By contrast, in Vf27 the stigmas were receptive previous to anthesis and without manipulation. Stigma receptivity of autofertile lines previous to anthesis was also reported by Chen [27], with the cuticle of the autosterile lines remaining intact even after anthesis while the autofertile ones were receptive in pre-anthesis [43]. Our results further confirm that stigma receptivity in autofertile lines is better synchronized with pollen availability since anthers dehisced prior to anthesis. On the contrary, autosterile lines showed a delayed stigma receptivity with more marked protandry, and therefore were less likely to find viable self-fresh pollen. Since pollen adhesion, hydration and germination is only possible when the stigmatic exudate has been released, the timing of rupture of the stigmatic cuticle is of high relevance for fertilization [28, 44]. We found that mean values for RUPTL and RUPTAREA in Vf27 were slightly higher than in Vf6, although the difference was not significant. Thus, our data do not confirm the earlier rupture of the stigmatic cuticle in the autofertile line, although previous experiments under similar conditions showed significant differences between parental lines (data not shown). This may be due to differences in manipulation or to an insufficient number of samples in the analysis.

Morphological traits such as length of flower, ovary and style showed higher values in line Vf6 than in Vf27. A larger style length in the autosterile line was previously reported by Kambal et al. [19], although Chen [27] reported the opposite result. Similar to other studies [19, 27], the style-ovary angle in the autosterile Vf6 was sharper than in the autofertile Vf27. Kambal et al. [19] hypothesized that sharper angles should block the ventral passage for pollen movement in autosterile lines, whereas the wider angles in the autofertiles should open this passage allowing the pollen to reach the stigma. On the other hand, style length as well as the style-ovary angle could also be involved in the mechanical fit between flowers and pollinators [45].

The stigma measures obtained from SEM photographs (STIGL, STIGAREA, NPAP, PAPL, PAPW, PAPD and STIGA) showed significant differences between parental lines. For all these traits, Vf6 showed higher values than Vf27, which could be explained by the larger size of the Vf6 flowers. However, mean papillae density based on counts of the same stigma area showed a higher density for Vf6 than Vf27. These results revealing higher density and longer papillae in autosterile than in autofertile lines are consistent with reports from Kambal et al. [19] and Chen et al. [27, 43]. Kambal et al. [19] also reported that autofertile lines had the tip of the stigma almost free of papillae, although these results were not supported by our analysis (data not shown).

In this work, several floral components related with autofertility were analyzed in the Vf6 x Vf27 faba bean RIL population. We also exploited prior phenotyping scores focusing on six traits that dissected the transformation of flowers into pods and ovules into seeds over several agronomic seasons and two different conditions, open field (allowing cross-pollination) and insect-proof cages (favouring autofertility). Our analyses revealed 19 QTLs related to pod and seed set, pollen quantity and size and floral and stigma morphological measures, co-localizing in six genomic regions, one for each faba bean chromosome.

The PS QTL in chr. III was associated with Vf_Ein4, a homologue of the histidine kinase EIN4 from *A. thaliana*. EIN4 is involved in ethylene synthesis in the stigma and style derived by pollination [46]. Ethylene causes the senescence of the perianth once pollination has been achieved. A strong signal of EIN4 in the locules of stamens, including the developing pollen and tapetum cells of *A. thaliana*, was reported by Hua et al. [47] highlighting a link between ethylene synthesis and pollen development. In chr. IV, the PS QTL was associated with Mtr1g106005(210), corresponding to a TUA4 tubulin alpha-4 chain protein related with cell structure and elongation in organ development [48]. This tubulin protein was also found in a proteomic analysis of the stigmatic exudate of *Olea europaea* [49], supporting a possible role in stigma structure and receptivity as well as fructification or pod setting. The QTL in chr. V is linked to two markers homologous to genes expressed in the floral stem of *A. thaliana*, tobacco, poplar, alfalfa and soybean [50]. Finally, marker Mtr4g088524 associated with a QTL of SS in chr. VI encodes a predicted homologue of the *Arabidopsis* ribosomal protein S6e (RPS6e) belonging to a family with multiple functions. Creff et al. [51] reported that a double RPS6 heterozygote (*RPS6A/rps6a, RPS6B/rps6b*) showed aborted ovules in the siliques. It is thus possible that RPS6e has a redundant function with other RPSs and our results could support this hypothesis.

For traits related with pollen size and production we found three regions with significant QTLs in chrs. II, V and VI. The QTLs in chrs. II and V are linked to markers MTR3g049400, identified as the glucose 6-phosphate/phosphate translocator 1/2 *GPT1/2* and MTR7g050950, which corresponds to the methylesterase *PMEPCRA*. *GPT1/2* is a key gene in lipid storage during pollen development and its loss of function reduces the accumulation of storage compounds, thus affecting pollen viability and germination [52, 53]. On the other hand, *PMEPCRA* functions in pectin synthesis and cell wall adhesion. The level of pectin esterification was related with proliferation and differentiation events during pollen development and pollen embryogenesis in *Capsicum annuum* L. [54, 55]. In our study, both markers were related to pollen quantity and quality traits (e.g. RATIO_SIZE, NORMAL%, TOTALS and NORMALQ), indicating a possible role of these genes in pollen development and final quantity. On the other hand, the two flanking markers of the QTLs in chr. VI are related to flower development and pod maturation, particularly with cellular compounds of flower buds [56].

Regarding morphological measures, we detected five regions with significant QTLs in chrs. I, III, IV and VI. The QTL in chr. I is associated with a RAPD marker of unknown function. In chrs. III and IV the flanking markers Vf_TT8 and MTR4g107940 are related to regulation of proline synthesis [57, 58], whereas the markers LOC109362751 and Mtr8g085280(81) in chrs. IV and VI are related with two genes implicated in transport and interaction with the plant hormones auxin and ABA [59, 60].

Finally, we detected a single significant QTL in chr. II for the STIGA trait. The flanking marker MTR1g102900 corresponds to a PPD1 TIFY domain/Divergent CCT motif family protein of *A. thaliana*. This gene promotes the early arrest of meristematic cell proliferation during organ development, including flower development [61]. The co-localization of two putative QTLs explaining the number of papillae and stigma length supports the function of *PPD1* during organ and flower development. Both pleiotropy and tightly linked genes could explain these QTL co-localizations.

## Conclusions

This is the first study attempting the localization of genetic determinants of pod and seed set in the faba bean genome by characterizing autofertility. We used a holistic approach recording morphological floral traits as well as pollen production and viability in the Vf6 x Vf27 RIL population. Data were further integrated with the QTL analysis of six autofertility traits dissecting the transformation of flowers into pods and ovules into seeds over several years and conditions. By combining QTL positions and functional information we identified six genomic intervals containing functional positional candidates controlling the trait. In the future, improved map resolution with higher density will further improve the results. We observed clustering of QTLs for different autofertility traits, with several linkage groups in chrs. I, II and V containing QTLs for flower and pollen traits that co-localized in the same positions. Interestingly, QTLs for flower and pollen measures in chrs. III, IV and particularly VI co-localize with QTLs for pod and seed set in insect-field cages. This is not surprising, given the often significant correlations among these traits and confirms that autofertility is a pleiotropic process in which an optimal value for a given trait can simultaneously influence the optima for other traits, a concept that needs to be considered particularly in marker assisted selection for autofertility. Therefore, our study adds new and important findings on the genomic locations controlling faba bean autofertility and strengthens the basis for molecular breeding strategies in this legume crop.

## Methods

### Plant material

A RIL population resulting from the cross of the faba bean parental lines Vf6 and Vf27 was used for the autofertility study. The population consists of 124 F8-F9 inbred lines, and has been used in previous studies to identify QTLs related to flowering time [62, 63], dehiscence [64], and other yield related traits. Line Vf6 is an asynaptic and highly autosterile equina type while Vf27 is highly autofertile paucijuga type, originated in India and considered to be close to the unknown wild progenitor of *V. faba* [65]. Seeds from these materials are conserved at the IFAPA germplasm bank.

### Field phenotypic evaluation

The evaluations were performed at IFAPA (Córdoba, Spain) along five agronomic seasons (2008–2009, 2009–2010, 2010–2011, 2012–2013 and 2014–2015). Assays were performed in two different environments: under insect-proof cages (years 2008–2009, 2009–2010 and 2014–2015) and/or in open field assays (2009–2010, 2010–2011 and 2012–2013). Cropping season 2009–2010 was the only where the RIL population was assayed in both environments. Ten seeds per RIL and parental line were sown in a complete randomized design with two repeats. A minimum of five plants per line and repeat were selected in each season in which ten nodes per plant were evaluated for the following traits: number of flowers per node (FN); number of pods per node (PN); the pod set (PS): number of pods/number of flowers x 100; ovules per ovary (OV); seeds per pod (SP) and seed set (SS): number of seeds/number of ovules x 100. OV, SP and SS were not evaluated in 2010–2011 and 2014–2015. The mean of the ten nodes for each trait was calculated for each plant.

Statistical analyses were performed in R v.3.6.1 [66], using the lme4 package [67]. To analyse differences between parental lines and environments, generalized lineal models were used using the pod and seed set as the response variable of binomial type (success = number of pods or seeds; fail = number of flowers-number of pods or number of ovules-number of seeds, respectively), and line and environment as fixed factors. Differences in pod and seed set at the population level were analysed with generalized linear mixed models (hereafter GLMMs) using the pod and seed set as the response variable of binomial type, year and environment as fixed factors and repeats as random effects nested within environment and year. GLMMs were finally also used to analyse differences between environments in 2009–2010 at the population level, using pod and seed set as a binomial response variable, environment as fixed factor and repeats as random effects nested within the environment. Unfortunately, this analysis could not be performed to test for differences between parental lines for this year due to missing data.

### Pollen size and pollen production quantification

Five flower buds of 16–17 mm were collected for each parental line and RIL and preserved in FAE (3.7% formaldehyde, 5% acetic acid, 50% ethanol) until samples could be analyzed. Flowers were dissected and the ten undehisced anthers were deposited in 1 ml of isotonic solution (Isoton II, Beckman, Fullerton, CA, USA) and sonicated for 30 min to facilitate pollen release. The sonicated pollen sample was then diluted into 50 ml of Isoton II and transferred to a particle counter (Coulter Multisizer III, Beckman Coulter, Miami, FL, USA), which provided estimates of the equatorial width of the pollen grains (hereafter pollen size) and the total number of pollen grains for a certain particle size (i.e. pollen production for each particle size). The size range established at the counter for the complete particle counting was 15–50 μm. Since the normal faba bean pollen size is estimated at ~ 30 μm [31], all particles between 27 and 38 μm were considered as normal pollen. All particles detected outside this range were classified as abnormal pollen. Finally, the following variables were considered: total pollen quantity in the range 15–50 μm (TOTALQ), pollen quantity with normal size (NORMALQ), percentage of normal pollen (NORMAL%), mean size of the pollen grains in the whole range (TOTALS), mean size of normal pollen (NORMALS) and finally the ratio of NORMALS divided by TOTALS (RATIO_SIZE). All measures were carried out at the Herbario service in Centro de Investigación, Tecnología e Innovación (CITIUS), University of Seville, Spain.

Differences between parental lines were analyzed with generalized linear models (GLMs) assuming Poisson errors (log link function) for the variables TOTALQ and NORMALQ, and binomial errors (logit link function) for NORMAL%. Models were fitted using quasi-binomial and quasi-Poisson errors due to over-dispersion of the data [68]. Pollen size was analysed with parametric (ANOVA, NORMALS) or no parametric tests (Wilcoxon’s test, TOTALS) according to the normality of the data. All analyses were carried out in R.

### Pollen viability and stigma receptivity

In order to know if pollen size and viability could be related, three plants from each parental line were sampled and for each of them, ten flowers of two different sizes (17–18 mm and 20–21 mm, five flowers each) were collected and immediately analysed. Three anthers per flower were randomly selected, crushed and stained with acetocarmine. The number of viable (dark red) and no viable (white or light red) pollen grains were counted in three fields of view and the pollen size measured under the microscope. A GLMM was performed to test for differences between parental lines using the pollen viability as the response variable of binomial type (success = number of viable pollen grains, fail = number of no viable pollen grains), line and flower size as fixed factors and fields of view nested within flower sample nested within plant as random effects. Differences in pollen size (log transformed) between lines and pollen type (normal vs. abnormal) were tested with a two way ANOVA analysis.

To test the stigma receptivity, ten flowers of each parental line were collected at four developmental stages (flower bud, pre-anthesis, complete anthesis and complete anthesis after a tripping treatment). Flowers were immediately dissected and the styles were included in a 0.2 µl vial containing distilled water and a piece of paper of Peroxtesmo KO (MACHEREY-NAGEL, Düren, DE). After ten minutes of incubation at room temperature, styles were observed under a stereoscopic microscope to identify colour change at the stigma surface which indicates the presence of peroxidases.

### Morphological measures of the flower, ovary and style

Ten flowers of 21–26 mm for each parental line and RIL were sampled and preserved in FAE until they could be analysed. Initially, a photograph of the flower was made to extract the flower length. Flowers were then dissected and a photograph of the pistil was made. The following measures were extracted from photographs using the software ImageJ [69]: flower length (FL), ovary length (OL), style length (SL), apex length (AL) and style-ovary angle (SOA) (Additional file 8). Besides, all these variables were standardised by flower length and included in the analyses. Since all variables were continuous, differences between parental lines were analysed with parametric or non-parametric tests depending on whether the variables were normally distributed or not.

### Stigma measures under the scanning electron microscope

Five to ten flowers per parental line and RIL were sampled and fixed in FAE. Flowers were dissected and ovaries were dehydrated in an increasing series of ethanol/water solutions from 50 to 100% of ethanol, then they were subjected to drying in a Leica EM CPD 300 machine. Samples were covered with gold and photographed under the SEM (scanning electron microscope JEOL JSM 6300 and JEOL JSM 7800 F), available at the Servicio Central de Apoyo a la Investigación (SCAI, University of Córdoba). The following measures were extracted from photographs: stigma length (STIGL), rupture length (RUPTL), percentage of rupture (%RUPT = RUPTL/STIGL x 100), stigma area (STIGAREA), ruptured area (RUPTAREA), percentage of ruptured area (%RUPTAREA = STIGAREA/RUPTAREA x 100), number of papillae on the STIGL (NPAP), papilla length (PAPL), papilla width (PAPW), papilla length/width ratio (PAP_LWRATIO), number of papillae per stigma length (NPAP/STIGL), papilla density (PAPD = number of papillas in 6 mm2 and stigma angle (STIGA) (see Additional file 9). ImageJ software was used to extract the measurements. The papilla length and width is the mean of three measures.

### QTL analysis

Using a previous linkage map [63], QTL detection was performed using the MapQTL v5.0 software [70]. The significant association between markers and traits was detected with the nonparametric Kruskal-Wallis test. Then, identification of putative QTLs in each linkage group (LG) was analysed by interval mapping test [71, 72]. In the multiple QTL analysis [73–75], only those markers significant at *P* = 0.01 were used as cofactors. Permutation analysis using 1000 replicates determined the QTL significance (p-value) [76], as implemented in MapQTL 5.0. Significant QTLs were established if their LOD is higher than the p-value. The QTLs confidence interval was represented using MapChart software [77]. The interval between LOD-1 and LOD-2 around the maximum LOD of QTL was defined as the support intervals. Finally, to identify the gene orthologs flanking each QTL, a BLASTp search against *Arabidopsis thaliana* protein database was performed.

## Supplementary Information

**Additional file 1. **Pod set and seed set values for parental lines in different environments.

**Additional file 2. **Percentage of viable pollen in Vf27 and Vf6 in two different flower sizes.

**Additional file 3. **Pollen samples of parental lines: (a) Vf6 and (b) Vf27, stained with acetocarmine. Viable pollen grains are stained in dark red whereas non-viable pollen is colorless. Bar: 50 µm.

**Additional file 4. **Results from two-way ANOVA analyzing the effect of parental line and pollen type (normal vs abnormal).

**Additional file 5. **Stigma staining with Peroxtesmo KO in different developmental stages. Upper line: Vf6; lower line: Vf27. Bar: 100 µm.

**Additional file 6. **List of non-significant QTLs detected in this study.

**Additional file 7. **Results of the BLASTp search in Arabidopsis thaliana (At), performed with the genes flanking the indicated faba bean QTL markers.

**Additional file 8. **Morphological measures of the flower. Ovary length (OL), style length (SL), apex length (AL) and style-ovary angle (SOA). Bar: 1 mm.

**Additional file 9. **Scanning Electron Microscope measures of the flower. a) Stigma length (STIGL), rupture length (RUPTL), stigma area (STIGAREA), ruptured area (RUPTAREA), papilla length (PAPL), papilla width (PAPW). b) papilla density (PAPD, number of papillas in 6 mm2). c and d) measure of stigma angle (STIGA). Bar: 200 µm.

## Data Availability

The data sets used and/or analysed during the current study will be available upon request to the corresponding author.

## Abbreviations

AL: apex length
FAE: formaldehyde - acetic acid - ethanol
FL: flower length
FN: flowers per node
GLMMs: generalized linear mixed models
GLMs: generalized linear models
NORMALQ: quantity of normal pollen
NORMALS: size of normal pollen
NORMAL%: percentage of normal pollen quantity
NPAP: number of papillas
OL: ovary length
OV: ovules per ovary
PAPD: density of papillae
PAPL: papilla length
PAPLWRATIO: ratio between PAPL and PAPW
PAPW: papilla width
PN: pods per node
PS: pod set
QTL: quantitative trait loci
RATIO_SIZE: ratio between normal pollen size and total pollen size
RIL: recombinant inbred line population
RUPTAREA: ruptured area
%RUPTAREA: percentage of ruptured area
RUPTL: ruptured length, %RUPT, percentage of rupture
SEM: scanning electron microscope
SL: style length
SO: seeds per ovules
SOA: style-ovary angle
SP: seeds per pod
STIGA: stigma angle
STIGAREA: stigma area
STIGL: stigma length
TOTALQ: total quantity of pollen
TOTALS: pollen size of the total range
