# Supplementary figures and images for: Study and QTL mapping of reproductive and morphological traits implicated in the autofertility of faba bean
Source: BMC Plant Biol. 2022 Apr 6;22:175. doi: 10.1186/s12870-022-03499-8 (PMC8985305; doi:10.1186/s12870-022-03499-8)

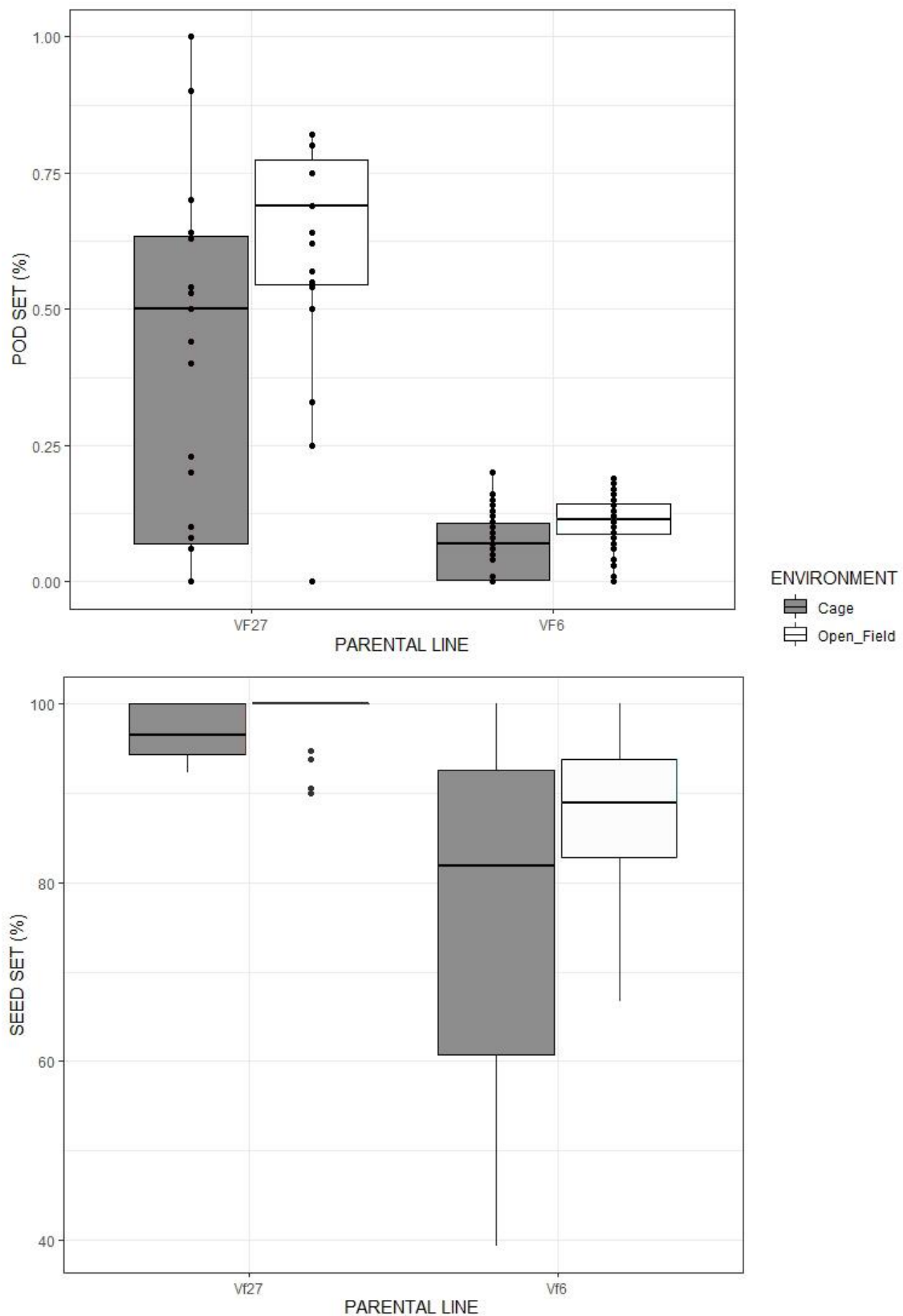

**Additional file 1.** Pod set and seed set values for parental lines in different environments.

Supplement: Supplementary file 1 — Additional file 1. Pod set and seed set values for parental lines in different environments. [file 12870_2022_3499_MOESM1_ESM.pdf]

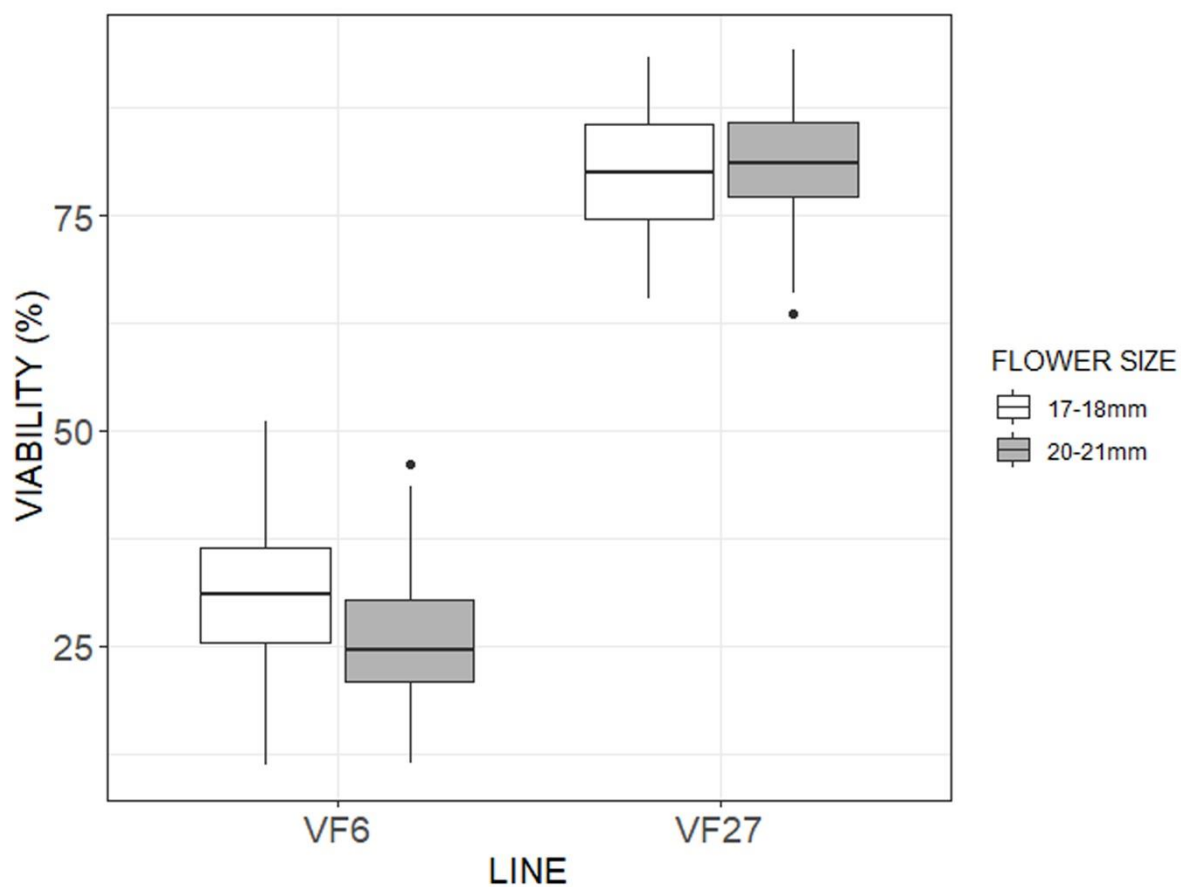

**Additional file 2.** Percentage of viable pollen in Vf27 and Vf6 in two different flower sizes.

Supplement: Supplementary file 2 — Additional file 2. Percentage of viable pollen in Vf27 and Vf6 in two different flower sizes. [file 12870_2022_3499_MOESM2_ESM.pdf]
