# Supplementary material for: Study and QTL mapping of reproductive and morphological traits implicated in the autofertility of faba bean
Source: BMC Plant Biol. 2022 Apr 6;22:175. doi: 10.1186/s12870-022-03499-8 (PMC8985305; doi:10.1186/s12870-022-03499-8)
Supplement: Supplementary file 3 — Additional file 3. Pollen samples of parental lines: (a) Vf6 and (b) Vf27, stained with acetocarmine. Viable pollen grains are stained in dark red whereas non-viable pollen is colorless. Bar: 50 µm. [file 12870_2022_3499_MOESM3_ESM.pdf]

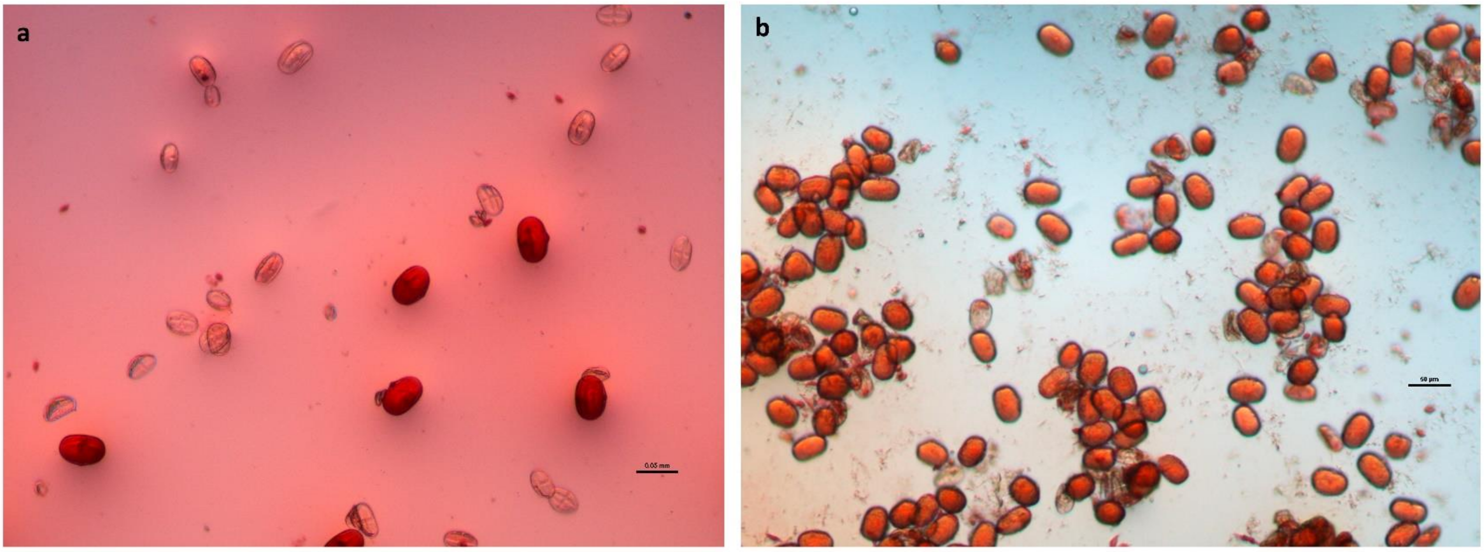


**Additional file 3**. Pollen samples of parental lines: (a) Vf6 and (b) Vf27, stained with acetocarmine. Viable pollen grains are stained in dark red whereas non-viable pollen is colorless. Bar: 50 µm.
