# Supplementary material for: Study and QTL mapping of reproductive and morphological traits implicated in the autofertility of faba bean
Source: BMC Plant Biol. 2022 Apr 6;22:175. doi: 10.1186/s12870-022-03499-8 (PMC8985305; doi:10.1186/s12870-022-03499-8)
Supplement: Supplementary file 4 — Additional file 4. Results from two-way ANOVA analyzing the effect of parental line and pollen type (normal vs abnormal). [file 12870_2022_3499_MOESM4_ESM.pdf]

Additional file 4. Results from two-way ANOVA analyzing the effect of parental line and pollen type (normal vs abnormal).

Source of variation	Df	Sum Sq	Mean Sq	F value	Pr(>F)
LINE	1	0.941	0.941	65.319	<0.001 ***
TYPE	1	2.479	2.479	172.134	<0.001 ***
LINE:TYPE	1	0.144	0.144	9.969	0.002 **
Residuals	116	1.671	0.014		
