# Supplementary material for: Study and QTL mapping of reproductive and morphological traits implicated in the autofertility of faba bean
Source: BMC Plant Biol. 2022 Apr 6;22:175. doi: 10.1186/s12870-022-03499-8 (PMC8985305; doi:10.1186/s12870-022-03499-8)
Supplement: Supplementary file 5 — Additional file 5. Stigma staining with Peroxtesmo KO in different developmental stages. Upper line: Vf6; lower line: Vf27. Bar: 100 µm. [file 12870_2022_3499_MOESM5_ESM.pdf]

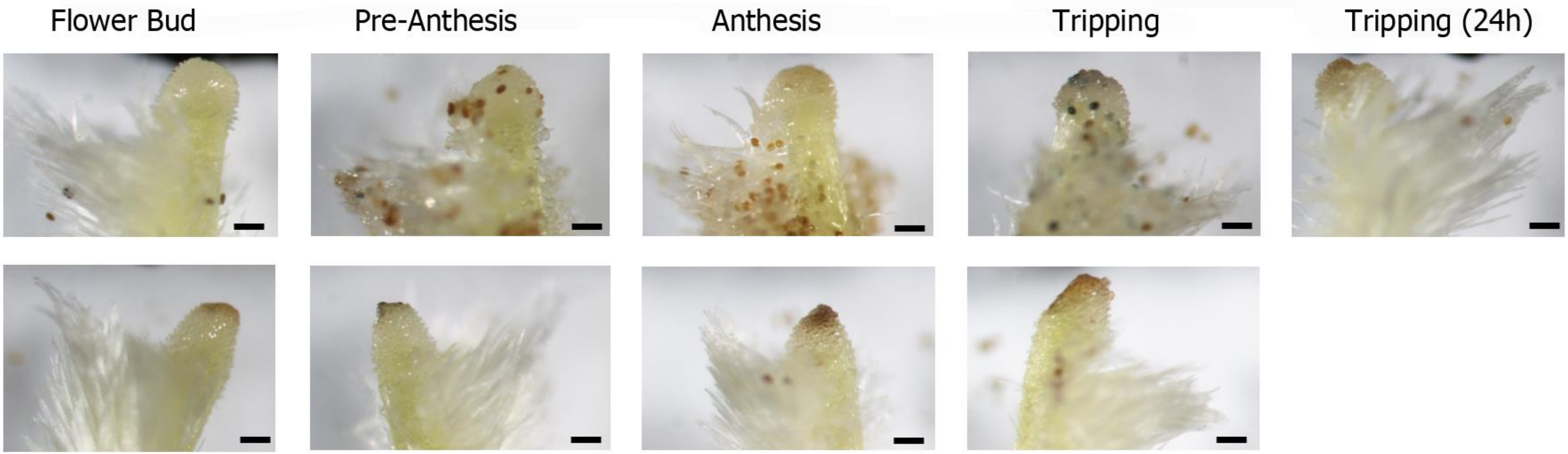

Additional file 5. Stigma staining with Peroxtesmo KO in different developmental stages. Upper line: Vf6; lower line: Vf27. Bar: 100 μm.
