# Supplementary material for: Study and QTL mapping of reproductive and morphological traits implicated in the autofertility of faba bean
Source: BMC Plant Biol. 2022 Apr 6;22:175. doi: 10.1186/s12870-022-03499-8 (PMC8985305; doi:10.1186/s12870-022-03499-8)
Supplement: Supplementary file 6 — Additional file 6. List of non-significant QTLs detected in this study. [file 12870_2022_3499_MOESM6_ESM.pdf]

Additional file 6. List of non-significant QTLs detected in this study.

Trait	Co-localized	Peak	Chr	Flanking Markers	LOD	Additive effects	R^2
SSR1_08-09_C*	**SL/FL(2)**	229,008	VI	MTR4g088595	2.71	-0.0717358	18.4
RATIO_SIZE(2)*	**OL(1)**	153,147	VI	OPK09_2	3.15	0.0396093	9.0
NORMAL%(2)*	**OL(1)**	153,147	VI	OPK09_2	3.00	907,106	8.7
SL/FL(3)*	**OL/FL**	417,609	I	OPJ09_4/OPL12_2	3.12	0.00293325	14.9
OL(2)*	**OL/FL**	428,395	I	OPL12_2	3.14	0.205966	12.7
AL*	**AL/FL**	229,589	III	OPJ14	2.44	-0.0310725	10.4
SOA/FL*	**NORMALQ(1)**	289,096	V	MTR7g050950	2.89	-0.0792338	11.2
SOA*	**NORMALQ(1)**	289,096	V	MTR7g050950	2.42	-147,345	8.8
OL/FL(2)*	**OL(1)**	149,245	VI	OPL18_2/OPK09_2	2.98	0.00799998	10.5
NPAP*	**STIGA**	825,007	II	OPJ09_5	2.36	0.387295	12.3
STIGL*	**STIGA**	824,007	II	OPJ09_5	2.99	654,215	10.7
