# Supplementary material for: Study and QTL mapping of reproductive and morphological traits implicated in the autofertility of faba bean
Source: BMC Plant Biol. 2022 Apr 6;22:175. doi: 10.1186/s12870-022-03499-8 (PMC8985305; doi:10.1186/s12870-022-03499-8)
Supplement: Supplementary file 7 — Additional file 7. Results of the BLASTp search in Arabidopsis thaliana (At), performed with the genes flanking the indicated faba bean QTL markers. [file 12870_2022_3499_MOESM7_ESM.pdf]

Additional file 7. Results of the BLASTp search in *Arabidopsis thaliana* (At), performed with the genes flanking the indicated faba bean QTL markers.

Traits	Chr.	Marker	At gene	Gene description	Query	E-value	Identity
RATIO_SIZE; NORMAL%; TOTALS	II	MTR3g049400	*AT1G61800*	GPT2 glucose-6-phosphate/phosphate translocator 2	89%	0.0	80.33%
			AT5G54800	GPT1 glucose 6-phosphate/phosphate translocator 1	99%	0.0	72.82%
STIGA	II	MTR1g102900	*AT4G14713*	PPD1 TIFY domain/Divergent CCT motif family protein	96%	4.00E-64	40.58%
PSR1_14-15_C	III	Vf_Ein4 (MTR1g079790)	*AT3G04580*	EIN4 Signal transduction histidine kinase, hybrid-type, ethylene sensor	96%	0.0	59.81%
AL/FL	III	Vf_TT8 (MTR1g072320)	*AT4G09820*	TT8 basic helix-loop-helix (bHLH) DNA-binding superfamily protein	76%	3.00E-102	72.14%
PSR2_14-15_C	IV	MTR1g106005(210)	*AT1G04820*	TUA4 tubulin alpha-4 chain	100%	0.0	95.78%
SL	IV	MTR4g107940	*AT5G62530*	ALDH12A1 aldehyde dehydrogenase 12A1	100%	0.0	80.22%
SL/FL	IV	LOC109362751	*AT2G26900*	BASS2 Sodium Bile acid symporter family	100%	0.0	78.15%
NORMALQ	V	MTR7g050950	*AT1G11580*	PMEPCRA methylesterase PCR A	98%	0.0	52.67%
PSR2_14-15_C	V	Vf_MTR7g112740	*AT3G05545*	RING/U-box superfamily protein	92%	3.00E-84	44.39%
PSR2_14-15_C	V	Vf_MTR7g118320	*AT5G54160*	OMT1 O-methyltransferase 1	97%	1.00E-136	51.84%
NORMALQ	VI	Mtr4g091610b	*AT1G13130*	Cellulase (glycosyl hydrolase family 5) protein	94%	3.00E-140	40.16%
TOTALS	VI	Mtr4g092820	*AT1G58290*	HEMA1 Glutamyl-tRNA reductase family protein	88%	0.0	70.79%
SSR1_09-10_C	VI	Mtr4g088524	*AT5G10360*	EMB3010 Ribosomal protein S6e	99%	3.00E-164	91.97%
SL/FL	VI	Mtr4g088595	*AT5G10360*	EMB3010 Ribosomal protein S6e	99%	2.00E-164	91.97%
SL	VI	Mtr8g085280(81)	*AT4G13750*	NOV Histidine nckinase-, DNA gyrase B-, and HSP90-like ATPase family protein	99%	0.0	43.03%
