# Supplementary material for: Study and QTL mapping of reproductive and morphological traits implicated in the autofertility of faba bean
Source: BMC Plant Biol. 2022 Apr 6;22:175. doi: 10.1186/s12870-022-03499-8 (PMC8985305; doi:10.1186/s12870-022-03499-8)
Supplement: Supplementary file 8 — Additional file 8. Morphological measures of the flower. Ovary length (OL), style length (SL), apex length (AL) and style-ovary angle (SOA). Bar: 1 mm. [file 12870_2022_3499_MOESM8_ESM.pdf]

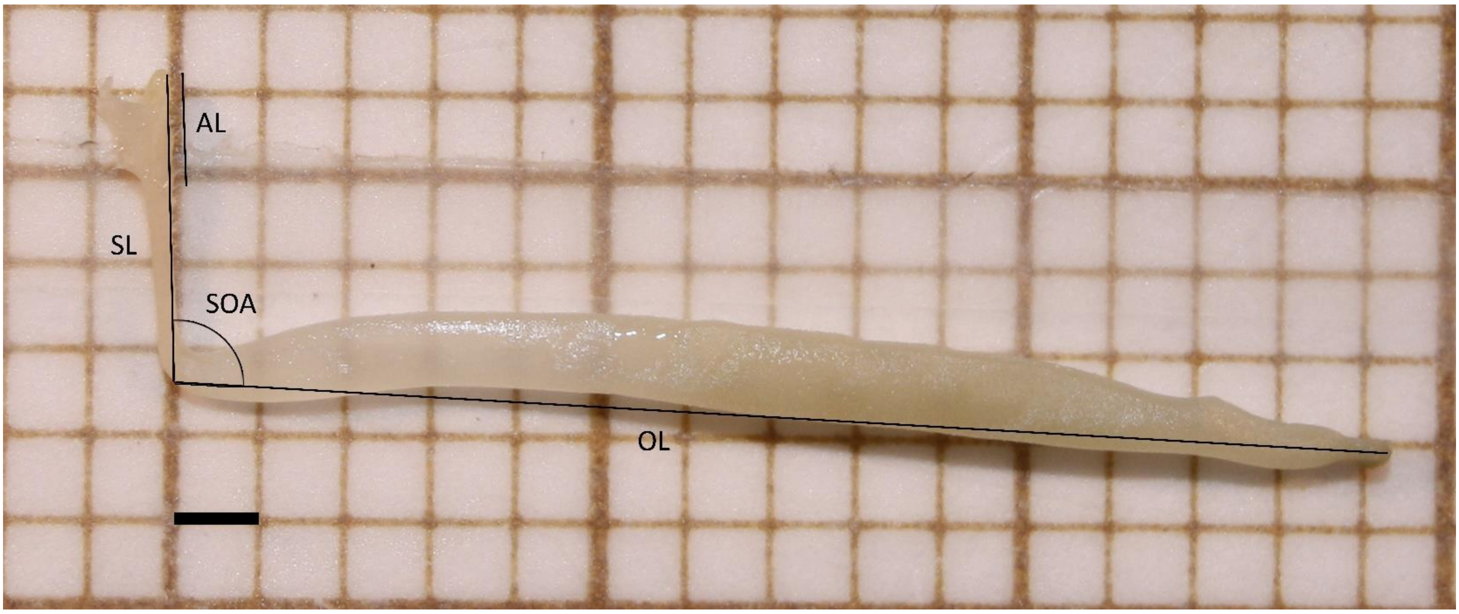

Additional file 8. Morphological measures of the flower. Ovary length (OL), style length (SL), apex length (AL) and style-ovary angle (SOA). Bar: 1 mm.
