# Supplementary material for: Study and QTL mapping of reproductive and morphological traits implicated in the autofertility of faba bean
Source: BMC Plant Biol. 2022 Apr 6;22:175. doi: 10.1186/s12870-022-03499-8 (PMC8985305; doi:10.1186/s12870-022-03499-8)
Supplement: Supplementary file 9 — Additional file 9. Scanning Electron Microscope measures of the flower. a) Stigma length (STIGL), rupture length (RUPTL), stigma area (STIGAREA), ruptured area (RUPTAREA), papilla length (PAPL), papilla width (PAPW). b) papilla density (PAPD, number of papillas in 6 mm2). c and d) measure of stigma angle (STIGA). Bar: 200 µm. [file 12870_2022_3499_MOESM9_ESM.pdf]

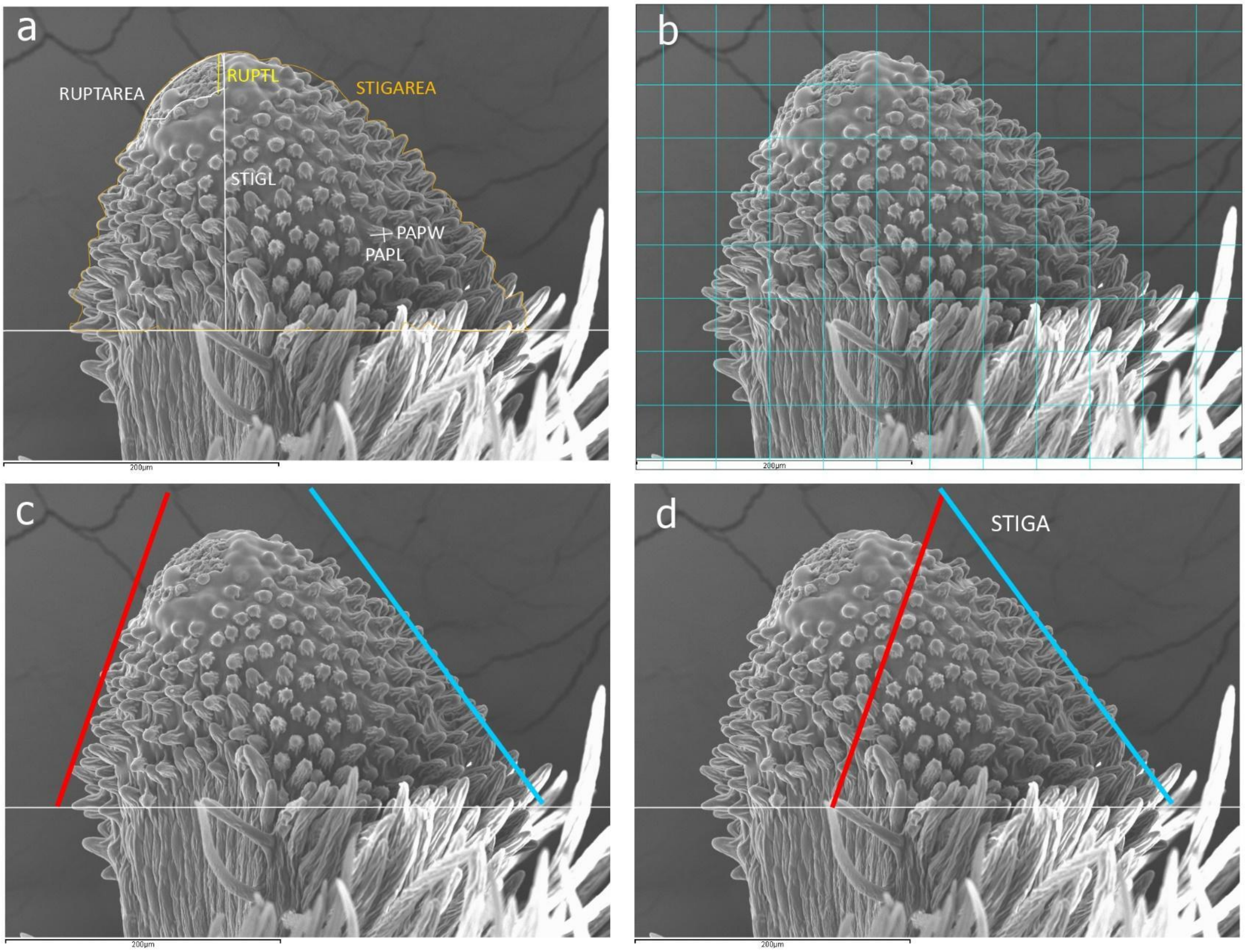

Additional file 9. Scanning Electron Microscope measures of the flower. a) Stigma length (STIGL), rupture length (RUPTL), stigma area (STIGAREA), ruptured area (RUPTAREA), papilla length (PAPL), papilla width (PAPW). b) papilla density (PAPD, number of papillas in 6 mm2). c and d) measure of stigma angle (STIGA). Bar: 200 µm.
